# SOULMATE: the Swedish study of liver transplantation for isolated colorectal cancer liver metastases not suitable for operation or ablation, compared to best established treatment—a randomized controlled multicenter trial

**DOI:** 10.1186/s13063-022-06778-9

**Published:** 2022-09-30

**Authors:** Veronica Reivell, Helga Hagman, Johan Haux, Carl Jorns, Per Lindnér, Helena Taflin

**Affiliations:** 1 Transplant Institute, Institute of Clinical Sciences, Sahlgrenska Academy at University of Gothenburg, Gothenburg, Sweden; 2 Sahlgrenska University Hospital, Gothenburg, Sweden; 3 Department of Oncology, Skanes University Hospital, Lund, Sweden; 4 Department of Oncology, Skaraborgs Hospital, Skovde, Sweden; 5 Department of Transplantation, Karolinska University Hospital, Stockholm, Sweden

**Keywords:** Randomized controlled trial, Colorectal cancer, Liver Metastases, Liver transplantation, Extended criteria donors

## Abstract

**Background:**

Around one fourth of patients with colorectal cancer present themselves with distant metastases at the time of diagnosis, and one additional one fifth of the patients will develop distant metastases during the disease, most commonly in the liver.

Surgical treatment such as liver resection or ablation, often combined with chemotherapy and targeted therapy, is the only treatment option with curative potential, but only about 20% of the patients with liver metastases are candidates for surgical intervention. Standard treatment for unresectable patients is palliative oncological therapy; however, less than 10% of these patients will achieve a 5-year survival.

Non-randomized studies indicate that liver transplantation could be an option for selected patients with colorectal liver metastases (CRLM), which are not suitable for operation or ablation due to surgical technical reasons such as massive tumor burden and small future liver remnant, or oncological reasons, for example, early relapse after liver surgery. Since there is a shortage of donated liver grafts, it is important to select the patient group that benefit most from the treatment. Although some studies present positive results from liver transplantation of CRLM, the results must be validated in a randomized controlled trial before this new indication for liver transplantation can be introduced as a clinical routine.

**Methods:**

The SOULMATE study is a randomized study evaluating if liver transplantation with liver grafts, primarily from extended criteria donors, increases overall survival in patients with CRLM, not suitable for resection or ablation, in comparison with best established treatment.

Patients will be randomized to liver transplantation (LT)+ best established treatment (BET) or to best established treatment only.

In the SOULMATE trial, we will evaluate the use of livers from extended criteria donors to decrease the risk of prolonging waiting time for patients on the waiting list for LT.

**Discussion:**

The SOULMATE study has the possibility to confirm the positive results of previous studies in a randomized setting. The use of extended criteria donors will make the results transferable globally, as most countries are struggling with organ shortage.

**Trial registration:**

Clinical Trial number: NCT04161092 registered 13 November 2019.

## Administrative information

Note: the numbers in curly brackets in this protocol refer to SPIRIT checklist item numbers. The order of the items has been modified to group similar items (see http://www.equator-network.org/reporting-guidelines/spirit-2013-statement-defining-standard-protocol-items-for-clinical-trials/).

**Table:** 

Title {1}	SOULMATE: The Swedish study of liver transplantation for isolated colorectal cancer liver metastases not suitable for operation or ablation; compared to best established treatment- a randomized controlled multicenter trial.
Trial registration {2a and 2b}.	Clinical Trials number NCT04161093
Protocol version {3}	The protocol version number is 1.3, dated: 2021-11-01.
Funding {4}	The SOULMATE trial has received financial support from the Swedish Cancer Society, The Swedish Medical Society and the Swedish state under the agreement between the Swedish government and the county councils and Region Vastra Gotaland.
Author details {5a}	1) Transplant Institute, Institute of Clinical Sciences, Sahlgrenska Academy at University of Gothenburg; Sahlgrenska University Hospital, Gothenburg, Sweden. 2) Department of Oncology, Skanes University Hospital, Lund, Sweden. 3) Department of Oncology, Skaraborgs Hospital, Skovde, Sweden. 4) Department of Transplantation, Karolinska University Hospital, Stockholm
Name and contact information for the trial sponsor {5b}	Professor Per Lindnér, Transplant Institute Sahlgrenska University Hospital, Gothenburg, Sweden. E-mail: per.lindner@surgery.gu.se
Role of sponsor {5c}	The SOULMATE trial is a sponsor driven academic trial The Transplant Institute at Sahlgrenska University Hospital is the sponsor and the coordinating center with three members in the trial steering committee. The trial steering committee consist of local study investigators. The study sponsor is responsible for administration of the study and collection and interpretation of data. The sponsor will also take a leading part in writing reports and submitting publication. Decisions will be taken in collaboration with all investigators in the study.

## Introduction

### Background and rationale {6a}

Colorectal cancer (CRC) is the third most common malignant disease in Sweden, as well as worldwide. It is also the fourth cause of cancer-related mortality globally [1].

Distant metastases are present at diagnosis (synchronous) in approximately 25% of all the patients, mainly in the liver (CRLM) [2], and about 20% of the patients will develop distant metastases within 5 years [3].

Surgical treatment, often combined with chemotherapy and biologic agents, is the only treatment options with curative potential [4].

Surgical case series have historically reported 5-years OS rates between 27 and 58% after hepatic resection [5–7]. Overall long-time survival after resection has radically improved the last decades, due to better staging, surgical technique, perioperative care, and systemic chemotherapy [8]. However, after initial liver surgery, up to 60% of the patients will have recurrence [9, 10], which demands reresections/and or ablations with acceptable outcomes for selected patients [11, 12]. Ablation provides an alternative in many patients with metastases of limited size with improved technique and low complication rates; however, for the majority of patients, surgical resection is still a gold standard [13–15].

Even if the limits of resectability have been extended over the last decades, only about 20% of patients with CRLM are candidates for surgical resection [16, 17]. The reasons could be associated with patient factors such as age, significant medical comorbidity, or anatomical, technical reasons such as proximity to vital anatomical structures and calculated insufficient future liver volume.

Patients with initially unresectable liver metastases can be offered upfront conversion chemotherapy to downstage the metastases to resectability. It is reported that between 12 and 33% of the patients who are initially assessed to have unresectable disease could have enough response to permit a complete (R0) resection, still with a high risk of recurrence [18, 19]. Standard treatment for unresectable patients is palliative chemotherapy, with the main goals to prolong survival and maintain quality of life. Recent advances enable tailored chemotherapy in combination with biological agents targeting angiogenesis or the epidermal growth factor receptor (EGFR) and have improved OS [20]. However, less than 10% of patients with unresectable CRLM will reach an OS of 5 years [21].

The procedure to resect all metastases, even those which are deemed to be unresectable, by total hepatectomy, followed by liver transplantation (LT), is already performed for other malignant indications, such as hepatocellular carcinoma (HCC), highly selected perihilar cholangiocarcinoma (CCC), and more recently for patients with very-early stage intrahepatic CCC. LT has also been performed for selected patients with liver metastases from neuroendocrine tumors [22–24]. In the beginning of the transplantation era, LT for unresectable CRLM was thought as a method for potential cure. According to the European Liver Transplant Registry, 58 patients underwent liver transplantation for unresectable CRLM 1977-1995 with a 5-year OS of 18%, and a graft loss of 44%, despite no signs of tumor recurrence [25].

In Vienna, a small series of LT for CRLM was performed between 1982 and 1994. Five year OS was reported as low as 12% with a 30-day mortality rate of 30% [26]. After these dismal results, unresectable CRLM was considered as a contraindication for LT. However, the fact that there were no standardized criteria for patient selection, no standardized immunosuppression protocols, and limited experience in the surgical and anesthesiologic procedure of LT might have contributed to the unsatisfactory results.

In 2006, the SECA-I study was opened at Oslo University Hospital. It was a groundbreaking prospective, non-randomized pilot study of LT for isolated nonresectable CLRM. The new protocol was possible to implement, since availability of liver grafts in Norway during the study period was sufficient. In the SECA-I study; 25 patients were included, of which four patients were dropouts due to extrahepatic disease. Finally, 21 patients underwent LT. All patients had undergone previous R0 resection for the primary CRC, no signs of extrahepatic disease, and had received at least 6 weeks of chemotherapy. The SECA-I study showed a 5-year OS of approximately 60%, even though there was recurrence of the CRC in 90% of the patients [27].

The main site of recurrence was in the lungs, but the lung metastases seemed to be slow-growing despite post-transplantation immunosuppression and did not significantly affect survival [28]. Compared to a similar matched cohort of isolated CRLM included in the first-line NORDIC-VII trial in which the efficacy of cetuximab in combination with fluorouracil/folinic acid and oxaliplatin was investigated, LT showed a significant increased OS [29].

In a Norwegian follow-up study, SECA-II, more restricted oncological criteria were used for inclusion, such as no lesion larger than 10 cm, 1 one-year span from CRC diagnosis before being accepted for LT, at least 10% radiological response to chemotherapy, and no sign of progressive disease at time of inclusion. An interim analysis of the first 15 patients showed an impressive 83% 5-year OS [30].

In 2017, a European retrospective study of 12 patients with nonresectable CRLM and LT were published [31]. There was no standardized patient selection, and no intervention protocol, but most of the patients had received chemotherapy. In this small study, the 5-year OS was 50%, and five patients were alive without signs of recurrence 108 months after LT.

The promising results from presented studies, in the light of organ shortage, show that a randomized control trial of LT as a treatment option in patients with CRLM not suitable for resection or ablation is warranted.

According to ClinicalTrials.gov, there are eight ongoing studies worldwide, assessing CRLM and LT.

We have identified five studies using liver grafts from deceased donors:

- The French/Belgian TRANSMET study NCT02597348 is a randomized study between palliative chemotherapy or LT. Eligibility criteria are similar to the SOULMATE study, although not the same focus on using extended criteria donors.
- The Spanish TRASMETIR study NCT04616495 is a prospective non-randomized cohort study. Inclusion criteria are among others lesions no larger than 5 cm and CEA not above 80 μg/l. The study population is 30 patients.
- The Italian COLT study NCT03803436 is a multicenter, prospective non-randomized study in which LT will be compared to a matched cohort enrolled in a parallel phase 3 study investigating triplet chemotherapy plus anti-EGFR. Patients with rectal cancer are not included.
- The Norwegian SECA III study NCT03494946 is a randomized controlled trial. Patients are all in a progressive status on chemotherapy and are then randomized between LT and other treatment that may include further chemotherapy, TACE, SIRT or other available treatment options. The patients will be randomized 1:1 to LT and chemotherapy/other treatment options. Sample size is 30 patients, and patients are allowed to have a limited lung metastases burden.
- The SECA II NCT01479608 will conduct a randomized controlled trial to explore whether liver transplantation in selected patients with liver metastases from CRC can obtain significant life extension and better health related quality of life compared to patients receiving surgical resection. The study has four arms:
-Arm A will include patients which are considered as resectable with limited metastatic burden.
-Arm B will include patients which are considerable as non resectable, with at least 10% response according to RECIST criteria on first line chemotherapy.
-Arm C will include patients with at least 10% response according to RECIST criteria on 2 or 3 line chemotherapy
-Arm D will include patients with expected overall survival of 6–12 months without a liver transplant. The patient might be included without further chemotherapy treatment and patients may have resectable pulmonary lesions at time of inclusion in the present study.

1. The experimental arm in the SOULMATE trial includes a liver transplantation which is a major surgical procedure. However, almost 200 liver transplantation are performed each year at the two transplant centers in Sweden, with a very low mortality rate. The clinical utility of liver transplantation for the individual with CRLM is thus the advantage of being offered a potentially curative treatment option with at least a 50% chance of disease-free survival at 1 year. A prolonged chemotherapy-free remission interval is beneficial compared to lifelong medical palliative treatment both for the patient and the health care system.

As the SOULMATE study is scheduled to use liver grafts from extended criteria donors, in addition to already established donor criteria, the study will contribute to valuable information not covered in other protocols.

The study has been approved by the Swedish Ethical Review Authority.

### Objectives {7}

The aim of this randomized study is to evaluate if liver transplantation in addition to best established treatment (BET) will extend OS for patients with CRLM not suitable for operation or ablation, compared to BET only. Secondary objectives include 2-year OS, median OS, and progression-free survival (PFS). Differences between the two groups regarding quality of life will be evaluated as well as health economic perspectives and the utility of biomarkers. Eventual risks by utilizing a broader donation pool will also be evaluated.

Primary objective:

- Five-year overall survival from randomization

Secondary objectives:

- Two-year overall survival from randomization
- Median overall survival
- Progression-free survival
- Hepatic progression-free survival
- Extrahepatic recurrence-free survival
- Quality of life
- Health economic evaluation
- Rate of primary non-function liver grafts
- Rate of donor-derived malignancies

### Trial design {8}

The SOULMATE trial is a prospective, multi-center, randomized controlled, open-label study. Patients will be followed actively according to the protocol for 5 years at each study center. It is expected that the percentage of subjects who reach the endpoint of overall survival after 5 years will be 55% in the study group and 10% in the control group. Based on this assumption, 45 subjects are planned to be randomized to the two treatment groups in a 5:4 ratio (LT to BET) to achieve 80% power for the superiority comparison (Fisher’s exact test) of the primary endpoint between the two treatment groups, with a 2-sided type I error of 5% and allowing for a 208% drop-out rate in the LT-group. An enrolment time of 60 months is expected.

## Methods: participants, interventions, and outcomes

### Study setting {9}

The SOULMATE trial will recruit patients from all Swedish hospitals included in the trial. All patients must be discussed at a national multidisciplinary conference. The study will only recruit Swedish citizens. The LT will be performed at the two Swedish liver transplant centers in Stockholm and Gothenburg.

### Eligibility criteria {10}

Inclusion criteria:

- Patients with liver metastases from colorectal adenocarcinoma, where it is not possible to achieve a radical resection with curative intention with a surgery and/or ablation. Ablation is here defined as radiofrequency ablation (RFA) or microwave ablation (MWA)
- Male or female 18 years or above
- Primary tumor removed with a standard oncological resection. Histologically verified adenocarcinoma from colon or rectum, with safe margins. Adequate TNM staging
- Liver metastases measurable by MRI or CT according to RECIST version 1.1 imaging within 4 weeks prior to inclusion
- No present or previous signs of extrahepatic metastatic disease or local recurrence according to
- MRI or CT of abdomen
- CT of thorax
- Whole body PET/CT scan

Previous resection of local relapse or non-hepatic metastasis more than 2 years ago can be accepted.

- A colonoscopy performed within the last 24 months in order to exclude existing CRC tumors
- ECOG performance status of 0 or 1
- Satisfactory blood tests: Hb ≥ 90 g/L (transfusions are permitted to achieve baseline hemoglobin level), white cell blood count > 3.0 × 10^9^/L, absolute neutrophil count (ANC) ≥ 1.5 × 10^9^/L, platelet level > 75, bilirubin < 2 ×upper normal level, ASAT, ALAT < 5 × upper normal level, calculated creatinine clearance ≥ 50 mL/min (MDRD)
- At least 2 months of first- or second-line chemotherapy for liver metastatic disease with PR or CR according to RECIST 1.1 or SD with a minimum of 10% relative decrease in sum of diameter of target lesions at any evaluation scan (taking as reference the baseline evaluation of the ongoing treatment line) (SD-10%). If the response is SD-10% at the first line, then it is necessary with SD-10% also at second line
- One year or more from the initial CRC diagnosis to the date of inclusion in the study
- Patient accepted for transplantation by a national study board
- Signed and dated written informed consent before the start of specific protocol procedures

Exclusion criteria:

- Pregnant or breast-feeding patients. Women of childbearing potential must have a negative pregnancy test performed within 7 days prior to the start of study
- Weight loss > 10% the last 6 months
- Other malignancies within the last 5 years, except CRC and low risk tumors such as basaliomas
- If a patient has pathological lymphatic nodules in the abdomen, a staging operation with PAD from the nodules with no signs of tumor cell involvement must be performed before inclusion
- BRAF mutations in primary tumor
- MSI-H in primary tumor
- Progressive disease (PD) at ongoing treatment line defined by RECIST 1.1
- Previous organ transplantation
- Liver metastases larger than 10 cm

Before randomization, eligible patients will undergo a preliminary, basic work-up to investigate, whether they are fit enough to undergo LT. Data will be assessed by a transplant hepatologist, and oncologist and potential study candidates will be discussed at the National Liver Surgery Multidisciplinary Conference. The national multidisciplinary team need to reach consensus that the patient fulfills the eligibility criteria for the study before the patient can be included in the study.

### Who will take informed consent? {26a}

Study nurse or treating surgeon will be obtaining the informed consent.

Informed consent must have been given voluntarily by each subject and signed by the patient and an investigator, before any study-specific procedures are initiated.

### Additional consent provisions for collection and use of participant data and biological specimens {26b}

The patients will also sign informed consent for use of clinical and biological data in ancillary studies.

### Interventions

#### Explanation for the choice of comparators {6b}

No comparator is used. Standard BET for unresectable patients is palliative chemotherapy often in combination with targeted therapy such as monoclonal antibodies. As the choice of drugs and other applicable anti-tumoral strategies is best tailored to each patient over time, the protocol does not specify best established treatment during the continuum of care for patients in the control group.

#### Intervention description {11a}

##### Arm A: liver transplantation (LT) + best established treatment (BET)

Patients subject to LT will, during the waiting time, receive individualized oncological therapy, with the aim to keep the disease stable but at the same time minimize side effects that could compromise transplantation.

If possible, patients randomized to LT should be transplanted within 12 weeks after randomization, although due to shortage of organs, waiting time might be longer. Hence, there is no upper time limit on the waiting list, if the patient fulfills the study criteria. The surgical procedure is performed as a standard liver transplantation.

The study patients included in the experimental arm will be added to the regular waiting list for LT. We will evaluate the use of livers from extended criteria donors to decrease the risk of prolonging waiting time for patients with already accepted indications for LT. While patients included in this study will have basically normal liver function with a normal MELD-score and no portal hypertension, the SOULMATE patients will probably be more able to tolerate an extended criteria liver graft, than patients with more suppressed liver function.

Examples of extended criteria donors are donors of higher age and/or higher grade of comorbidity, such as obesity/liver steatosis and donors with a risk of transmission of infectious or malignant disease.

The guide to the quality and safety of organs for transplantation (EDQM 2016, 29) will be used. Donors with a high risk for transmission of malignancy can be accepted by the transplant surgeon after consideration in individual cases, but donors with an unacceptable risk for transmission defined according to EDQM (29) will not be accepted.

In case of an early technical complication or delayed graft function in a study patient, with a need for an early re-transplantation, a kind request for a liver could be applied as for other liver transplanted patients. If such a complication occurs in the study, a thorough analysis of the causes is mandatory.

##### Immunosuppressive management

After LT patients will initially receive the standard immunosuppressive protocol at the Sahlgrenska University Hospital, which consists of basiliximab and steroids for induction, mycophenolate mofetil (MMF), and tacrolimus (Tac) for maintenance immunosuppression. MMF should be exchanged to Everolimus (mTOR-inhibitor) an immunosuppressive drug, which also have presented anti- tumor effects [32], but this must be done at the earliest 4 weeks postoperatively.

The reason for using Tac as immunosuppression in the initial period of the transplantation is the reported issues of wound healing problems and occurrence of hepatic vessel events with mTOR treatment. Patients will also receive standard anti-infective prophylaxis during six months, including sulfamethoxazole-trimethoprim and valganciclovir.

##### Arm B: best established treatment

The treating physician at the oncology clinic will, together with the patient, decide the treatment. All applicable antitumoral treatment strategies, as well as other experimental treatments, are accepted in the study; however, no cross-over to arm A will be allowed.

Standard BET for unresectable patients is palliative chemotherapy often in combination with targeted therapy such as monoclonal antibodies. As the choice of drugs and other applicable anti-tumoral strategies is best tailored to each patient over time, the protocol does not specify best established treatment during the continuum of care for patients in the control group.

#### Criteria for discontinuing or modifying allocated interventions {11b}

If a patient during the waiting time in arm A has extrahepatic progression, proceeding to LT is not allowed. If a patient progress only within the liver, it is possible to stay on the waiting list for LT, until the patient is proclaimed technically not transplantable by the liver transplant team.

Patients may be discontinued from the study at any time if, in the opinion of the investigator, it is medically necessary or if it is the expressed wish of the patient. Patients are free to discontinue their participation in the trial at any time.

#### Strategies to improve adherence to interventions {11c}

Patients will be followed by treating physicians in both arms. No specific interventions will be performed outside clinical praxis.

#### Relevant concomitant care permitted or prohibited during the trial {11d}

No relevant concomitant care will be prohibited during the trial. In arm A, modification of the chemotherapy treatment might be initiated when a patient is active on the transplantation waiting list, in order to minimize risk of adverse events which might jeopardize transplantation.

#### Provisions for post-trial care {30}

No provision for post-trial care will be offered.

#### Outcomes {12}

The primary endpoint is to evaluate if the addition of liver transplantation will increase overall survival for patients with isolated colorectal liver metastasis not suitable for operation or ablation, compared to best established treatment, measured as 5-year OS from randomization.

Secondary objectives include 2-year OS, median OS, and PFS, described as hepatic and extrahepatic recurrence respectively. We will also evaluate if there are any differences between the two groups when it comes to quality of life, measured according to EORTC-QLQ C30, 1 and 2 years after inclusion. Furthermore, we will evaluate if there are any differences regarding health economic perspective, evaluated by quality-adjusted life years (QALYs). The rate of primary non-function liver grafts and donor-derived malignancies will be recorded and reported as SAEs (serious adverse events).

In addition, major challenges in curative treatment programs for CRLM are:

- Early identification of subclinical disease not visible by standard imaging
- Identification of predictive markers for response to adjuvant chemotherapy
- Early detection of treatment failure/localized recurrence

To meet these challenges, the program also includes research to define novel diagnostic and prognostic factors. The study cohort will provide a unique opportunity to study histological and molecular differences between the primary tumor and subsequent metastases. The patients will all have undergone surgery and oncological therapy in a monitored fashion, thereby giving rise to a well-defined cohort. Plasma and tissue will be collected for the study of dynamic biomarkers during oncological treatment and during immunosuppression in the transplanted group.

#### Participant timeline {13}

**Fig. 1:**
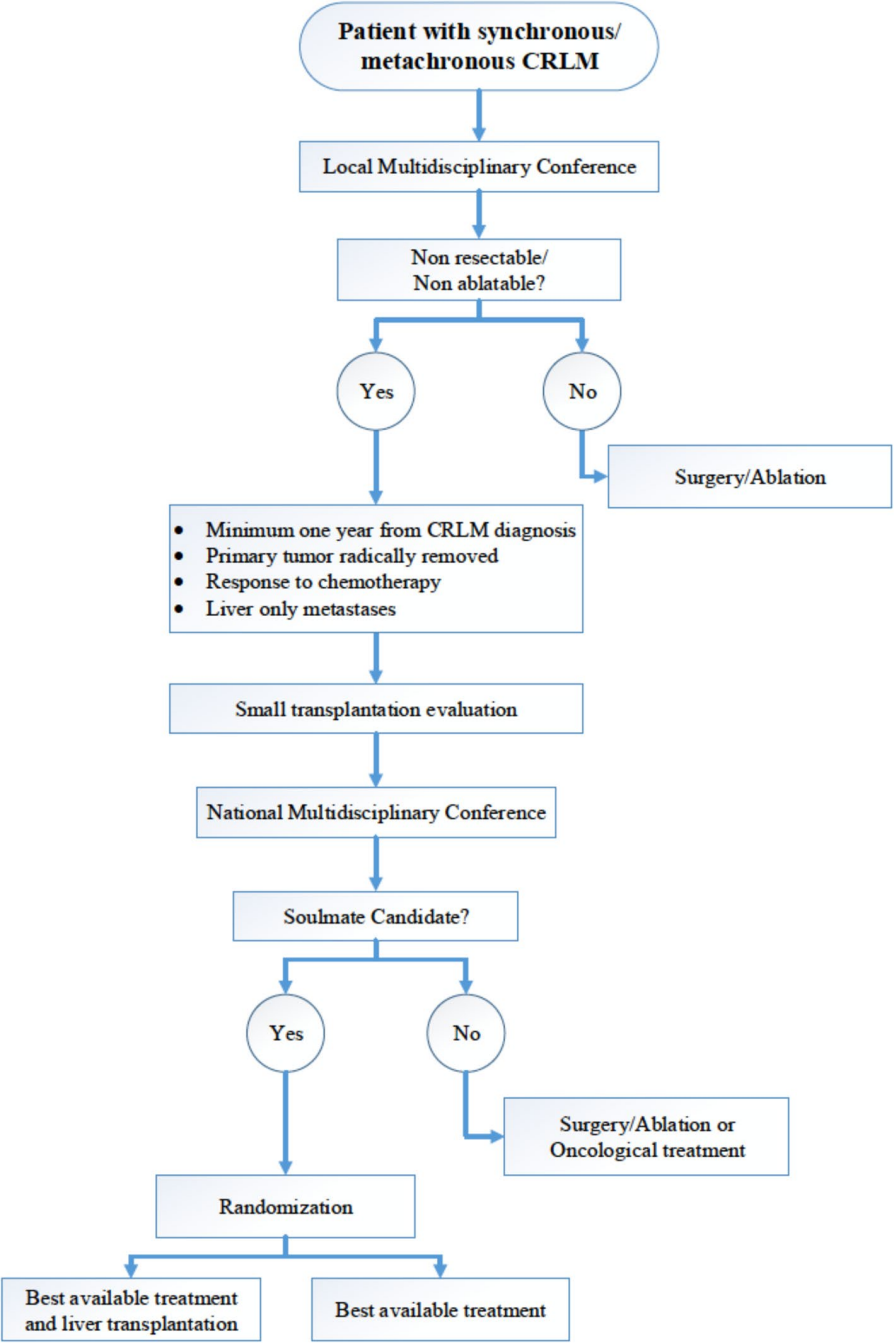
Flow chart for inclusion of patients in SOULMATE

**Fig. 2:**
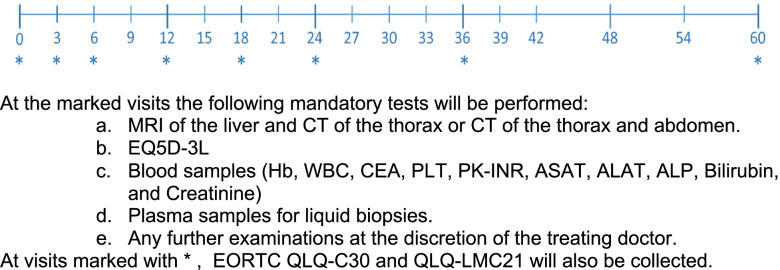
Protocol for study visits after inclusion in the SOULMATE trial

Timeline is presented in Figs. 1 and 2.

#### Sample size {14}

It is expected that the percentage of subjects who will reach the endpoint of overall survival after 5 years will be 55% in the study group and 10% in the control group. Based on this assumption, with 80% power for the superiority comparison (Fisher’s exact test) of the primary endpoint between the two treatment groups, and a 2-sided type 1 error of 5%, allowing for a 20% drop-out rate in the LT-group, we plan to randomize 45 subjects to the two treatment groups in a 5:4 ratio (LT:BET).

#### Recruitment {15}

Information about the study and the possibility to enroll patients will be communicated on national meetings with attending oncologists as well as colorectal and liver surgeons. Information has been communicated to the Swedish Colorectal Patients Associations and through the National Swedish Medical Journal. An enrolment time of 60 months is expected.

### Assignment of interventions: allocation

#### Sequence generation {16a}

The patients will be enrolled by the physicians at respective sites when the subjects have been accepted by the National Liver Surgery Conference. The randomization procedure will be computer-generated through the electronic case report formula (MediCase eCRF), and it is not possible for physicians to predict the outcome of the randomization. Stratification will be performed per included site; the limited size of the study allowed for no other stratification factors.

#### Concealment mechanism {16b}

Randomization is done by the eCRF only and concealed from physicians until the intervention is allocated.

#### Implementation {16c}

After the subjects have been assigned to the study or control group through the randomization procedure, they be informed by their treating physician. Patients subjected to the study group will then be referred to one of the two transplant center for pre-operative work-up.

### Assignment of interventions: blinding

#### Who will be blinded {17a}

The SOULMATE trial is an open trial, and no blinding will be used in any part of the study procedures.

#### Procedure for unblinding if needed {17b}

The design of the SOULMATE trial is open label, so unblinding will not occur.

### Data collection and management

#### Plans for assessment and collection of outcomes {18a}

The patients will be followed for five years. Study visits will be performed at each center at baseline and every third month the first 3 years and every sixth month hereafter. Follow-up will include the following mandatory tests: MRI or CT of the liver and CT of the thorax, blood samples, including tumor-markers and plasma samples for liquid biopsies. European Quality of Life Five Dimension Three Level Scale (EQ5D-3L) quality of life questionnaire will be answered at every visit, and EORTC QLQ-C30 and QLQ- LMC21 will be filled in at baseline and at 3, 6, 12, 18, 24, 36, and 60 months.

The timeline is to be found in an electronic clinical report form (e-CRF).

#### Plans to promote participant retention and complete follow-up {18b}

All study subjects, except those that withdraw consent for the study, will be followed for the study period. Patients that discontinue their follow-up visits, without withdrawing consent, will be followed for survival.

#### Data management {19}

The designated site staff will enter the data required by the protocol into the e-case report forms (eCRF). The principal investigator at each participating site is responsible for assuring that data entered into the eCRF is complete, accurate, and that entry is performed in a timely manner. The signature of the investigator will attest the accuracy of the data on each eCRF. If any assessments are omitted, the reason for such omissions will be noted on the eCRFs. Corrections, with the reason for the corrections, will also be recorded/tracked in the eCRF. All data management activities will be completed prior to final closure of the database. All original research data will be managed and stored according to Swedish law.

#### Confidentiality {27}

All patient data collected and processed for the purposes of this study will be managed by the sponsor with adequate precautions to ensure the confidentiality of those data, and in accordance with applicable national and national laws and regulations on personal data protection. No patient-identifiable data will be obtained. In any presentations of the results of this study, at meetings or in publications, the patients’ identity will remain confidential. In all activities, the GDPR (General Data Protection Regulation) will be followed to ensure protection of sensitive personal information, and the study will perform in accordance with WHO guidelines for good clinical practice (GCP).

#### Plans for collection, laboratory evaluation, and storage of biological specimens for genetic or molecular analysis in this trial/future use {33***}***

Plasma samples will be collected during the study period at every study related visit. In patients randomized to liver transplantation, tissue from metastases as well as macroscopically normal liver will be sampled. After preparation and aliquoting, both the plasma and the tissue samples will be stored in – 70 °C, in a preexisting biobank at the Sahlgrenska University Hospital.

### Statistical methods

#### Statistical methods for primary and secondary outcomes {20a}

The Transplant Institute, Gothenburg, will be responsible for all statistical programming and analysis, as well as quality control and validation of programming and statistical analysis. The responsible biostatistician will coordinate the statistical analysis. A detailed description of all the statistical analyses of all efficacy and safety variables together with an overview of tables and figures will be given in a separate statistical analysis plan (SAP). The SAP has been finalized before the database of the study is locked. Any deviations from the SAP will be justified in the clinical study report.

The primary analysis of efficacy data will be based on the intention-to-treat (ITT) population, defined in the SAP. Patients will be analyzed according to their randomized treatment.

#### Interim analyses {21b}

A data monitoring committee (DMC) will be appointed. The DMC will perform an interim analysis after 40% of the subjects in the study have been followed for 2 years. The sponsor has the right to terminate or change the trial prematurely if there are any relevant medical or ethical concerns or if completing the trial is no longer feasible. If such action is taken, the reasons for terminating the trial must be documented in detail. Premature termination of the trial will be considered if:

- The risk-benefit balance for the trial subjects changes markedly
- The sponsor considers that the trial must be discontinued for safety reasons
- An interim analysis or results of other research show that one of the trials treatments arms are superior or inferior to another

Inclusion in the study will be temporarily stopped if two post-operative deaths within 90 days of liver transplantation occurs. The causes of death will then be analyzed in detail and discussed within the data monitoring committee (DMC) before any final decision about continuing or stopping the trial.

In the case of a prematurely stopped trial, the patients will be taken care of and followed at the discretion of the treating physician.

If, during the conduct of the study, new publications clearly shows that liver transplantation is beneficial for the study population, the study protocol will be amended so that all consecutively included patients are assigned to the transplantation arm.

#### Methods for additional analyses (e.g., subgroup analyses) {20b}

In the liver transplantation group the overall survival after 2 and 5 years from randomization will be analyzed in the following subgroups:

- Subjects transplanted with a liver from a donor with a history of malignancy will be compared to subjects transplanted with a liver from a donor with no history of malignancy
- Subjects transplanted with a liver from a donor previously tested positive for virus (hepatitis or HIV) compared to subjects transplanted with a liver from a donor without positivity for virus
- Subjects that have experienced an extrahepatic recurrence will be compared with subjects that has experienced an intrahepatic recurrence
- Subjects that have undergone surgical or ablative treatment for recurrence will be compared to subjects that have not undergone any surgical or ablative treatment for recurrence

#### Methods in analysis to handle protocol non-adherence and any statistical methods to handle missing data {20c}

The data will be analyzed both for intention to treat and per protocol. In the intention-to-treat analysis, all subjects will be analyzed according to the arm they have ben randomized to. Patients in arm A, which have been assigned to liver transplantation, will be analyzed in this group even if they for any reason had not undergone liver transplantation. In the per-protocol analysis; the subgroup of patients that have been assigned to liver transplantation but not undergone transplantation will be excluded from the analysis.

Since all patients will be under continuously supervision by treating medical staff, most frequently oncologist, the risk of missing follow-up is considered low. For the primary outcome, survival, the amount of missing data is expected to be small. For parameters with a higher proportion of missing outcomes per group, the characteristics of participants for whom no outcomes were observed, and the reasons for missing outcome data will be reported.

#### Plans to give access to the full protocol, participant level-data and statistical code {31c}

The protocol is available for the public, but dataset and statistical codes will not be made publicly available to protect confidentiality of the participants.

### Oversight and monitoring

#### Composition of the coordinating center and trial steering committee {5d}

The coordinating center has coordinating research nurses as well as a principle investigator for day-to day support. The trial steering committee consists of both oncologist and surgeons from multiple study sites.

#### Composition of the data monitoring committee, its role and reporting structure {21a}

A data monitoring committee (DMC) is appointed consisting of clinicians and a biostatistician that are independent of the trial. The DMC will perform the interim analysis where they will analyze safety of the trial as well as efficacy of ASA compared to placebo. A clinical study monitor will visit all investigating sites on a regular basis. The monitor will review the relevant CRFs for accuracy and completeness and will ask the site staff to adjust any discrepancies as required. The monitor is independent from the sponsor.

#### Adverse event reporting and harms {22}

Serious adverse events (SAE) will be reported for both treatment arms according to the GCP guidelines. Primary non-functioning of liver grafts and donor-derived malignancies could be attributed to the utilization of marginal liver grafts and should be reported as SAEs. Adverse events will not be reported in this study.

#### Frequency and plans for auditing trial conduct {23}

The sponsor is responsible for monitoring the clinical study to ensure that subjects’ human rights, safety, and well-being are protected and that the study is properly conducted in adherence to the current protocol, that study data reported by the investigator/sub-investigator are accurate and complete, and that they are verifiable with study-related records such as source documents. The sponsor has assigned an independent study monitor for proper monitoring. The monitor will conduct an initiation visit to each site when the first patient has been included at the site and will then monitor the patients in the study in accordance with the monitoring plan.

#### Plans for communicating important protocol amendments to relevant parties (e.g., trial participants, ethical committees) {25}

Any changes to the study, which arise after approval of the protocol, will be documented as protocol amendment or administrative amendments. Depending on the nature of the amendment and/or revision, either approval or notification is required from the Ethical Review Agency. The changes will be effectuated only after the approval of the sponsor and the Ethical Review Agency (if applicable).

Written verification of the Ethical Review Agency approval will be obtained before any amendment is implemented.

#### Dissemination plans {31a}

Upon study completion and finalization of the study report, the results of this study will either be submitted for publication and/or posted in a publicly assessable database of clinical study results. The results of this study will also be submitted to the Competent Authority and the Ethics Committee according to EU and national regulations. All personnel who have contributed significantly with the planning and performance of the study (Vancouver convention 1988) may be included in the list of authors.

## Discussion

For CRC patients with isolated liver metastases not suitable for operation or ablation, the best-established treatment will yield a 5-year OS of only around 10%. A treatment option that increases long time survival for this group is therefore of great need.

There are reasons to believe that liver transplantation could prolong life considerably compared to palliative chemotherapy (24-26), but to provide solid evidence for this, a RCT is warranted. The SOULMATE study will also provide information if adding CRC liver metastasis not suitable for resection or ablation for liver transplantation is defendable with the current shortage of organs.

There are currently two ongoing randomized studies that evaluate liver transplantation with deceased donors, as an option in patients with liver metastases from colorectal cancer, not possible to resect or ablate.

The French-Belgian study, TRANSMET, which is the study most similar to the SOULMATE trial has in our opinion, together with SOULMATE, the best opportunity to prove the value of LT as a treatment of CRLM in a randomized way. In the ongoing Norwegian study, SECA III, the study population will have more advanced disease than the populations in TRANSMET and SOULMATE.

Neither of these studies are investigating whether transplantation of patients with CRLM is feasible in a population where organs for transplantation are a scarce resource, as in Sweden. With a broader spectrum of indications for liver transplantation, the recipients and the needs for liver grafts will exceed the donor pool. In the SOULMATE study, liver grafts from extended criteria donors will be preferred to use for LT. Therefore, the study reflects more a real-life situation, with organ shortage. We will also have the possibility to evaluate risks with extended donors in general.

The decision to use organs with a higher risk of transmission of malignancies and maybe a higher risk for delayed or primary non-function grafts can be justified as we consider the risk to be much less than the expected survival benefits. All patients included in the study have a situation with metastatic cancer and poor prognosis with conventional treatment and the possible advantages of improved survival are considered to outweigh the risks. The patients will not suffer from liver failure with portal hypertension, which probably make them more likely to tolerate a graft with extended criteria compared to other patients on the waiting list for LT.

A cost-benefit analysis from SECA I suggested that liver transplantation was cost-effective but only for highly selected patients as included in our protocol (27). A limitation of the SECA I report is that the trial population was small, with no control group, and follow-up was relatively short. A cost-benefit analysis will also be performed in our randomized study, as well as evaluation from the patient´s perspective of quality of life.

Our study could face the risk for a slow recruitment due to lack of donors and recipients. To investigate how many patients that could fulfill the study eligibility criteria, patients that were discussed at the multidisciplinary team conference in the Stockholm area 2013–2018 were investigated. We found fewer possible recipients for liver transplantation than expected, but the eligibility criteria were set to meet the best outcome according to characteristics found in the Norwegian SECA-studies and not to the specific criteria in the SOULMATE study. Many potential recipients were excluded because of remaining primary tumor [33].

In conclusion, the Swedish SOULMATE-study has the possibility to confirm the positive results of the Norwegian SECA studies in a randomized way. The use of extended criteria donors will improve the possibility of transferring the results in countries like Sweden, which struggle with an organ shortage, making it difficult to expand current liver transplant indications.

### Trial status

The protocol version number is 1.3, dated: November 1, 2021. The study started to enroll patients in December 2020. The recruitment will approximately be completed in December 2027.

## Abbreviations

BET: Best established treatment
CRLM: Colorectal liver metastases
LT: Liver transplantation
SAE: Serious adverse event
AE: Adverse event
DMC: Data monitoring committee
OS: Overall survival
